# Demographic and socioeconomic factors influencing ADHD diagnosis in children and adolescents: a population-based nested case–control study

**DOI:** 10.1007/s00787-026-03007-5

**Published:** 2026-03-16

**Authors:** Olast Arrizibita, Marta Gutiérrez-Valencia, Luis Carlos Saiz, Arkaitz Galbete, Leire Leache, Juan Erviti, Julián Librero

**Affiliations:** 1 Dept. of Statistics, Computer Science and Mathematics, Public University of Navarre, Pamplona, Spain; 2 NNBi, Pamplona, Spain; 3 Innovation and Organization Unit, Navarre Health Service, Pamplona, Spain; 4 Navarre Institute for Health Research (IdiSNA), Pamplona, Spain; 5 Institute of Smart Cities, Public University of Navarre, Pamplona, Spain; 6 Pharmacy and Services Sub-Directorate, Navarra Health Service, Pamplona, Spain; 7 Methodology Unit, Navarrabiomed-HUN-UPNA, Pamplona, Navarra Spain; 8 Research Network on Chronicity, Primary Care and Health Promotion (RICAPPS), Madrid, Spain

**Keywords:** Attention deficit hyperactivity disorder, Children and adolescents, Socioeconomic factors, Transients and migrants, Schools, Educational status, Case-control studies

## Abstract

Attention deficit hyperactivity disorder (ADHD) is among the most commonly diagnosed psychiatric disorders in childhood, with an estimated prevalence in children and adolescents of 5.9% in Navarre (2019) and 6.8% in Spain (2012). However, demographic and socioeconomic factors influencing diagnosis remain insufficiently explored. The aim was to examine associations between demographic factors (month of birth, migration status, number of siblings) or socioeconomic variables (household income, residence type, school type) and ADHD diagnosis in children and adolescents. Population-based case-control study nested in a cohort of individuals born between 1991 and 2011 and followed from 2007 to 2019 using linked health and educational records from Navarre, Spain. Incident ADHD cases were age and sex-matched to three controls (1:3). Data were analyzed with multivariate conditional logistic regression. Lower family income (< €18,000/year) was associated with a 23% increased risk of ADHD diagnosis (OR = 1.23, 95%CI: 1.15–1.31). Large sized rural areas showed less likely diagnostic rates compared to urban residence (OR = 0.88, 95%CI: 0.82–0.93). The month of birth significantly influenced diagnosis likelihood, with those born later in the year showing increased odds, peaking at 69% higher odds in October-December (OR = 1.69, 95%CI: 1.55–1.83). Having ≥ 3 siblings reduced diagnosis likelihood by 17% compared to only children (OR = 0.83, 95%CI: 0.76–0.91). Migration status and school type interaction analysis revealed that migrant children in public schools had significantly lower ADHD diagnoses compared to non-migrant peers; this difference diminished in charter schools. This study confirms, in a large population cohort, the significant impact of socio-demographic factors on the likelihood of ADHD diagnosis, such as socio-economic status, month of birth, migrant status, number of siblings, and place of residence, and provides new insights into the role of school type and the interaction between school type and migrant status, this interaction representing a relatively understudied factor. These associations highlight disparities and potential diagnostic biases. Strategies to address these inequalities are essential for consistent ADHD diagnostic practices and equitable healthcare interventions.

**Supplementary Information:**

The online version contains supplementary material available at 10.1007/s00787-026-03007-5.

## Introduction

 Attention deficit hyperactivity disorder (ADHD) is one of the most commonly diagnosed psychiatric disorders in childhood and adolescence, and is associated with difficulties in academic performance, social relationships, and quality of life. Epidemiological studies estimate an expected prevalence of ADHD of around 5–7% in children and adolescents; a recent meta-analysis placed this prevalence at 7.6% in children aged 3–12 years and 5.6% in adolescents aged 12–18 years [1]. In Spain, a systematic review and meta-analysis of population studies estimated an overall prevalence of 6.8% in children and adolescents in 2012, although with notable regional and methodological heterogeneity [2]. As for the province of Navarre, the most recent estimate comes from 2019, showing an ADHD prevalence of 5.9% [3]. However, the prevalence of registered diagnoses is considerably lower: it is estimated that only 2% to 2.5% of children and adolescents in Spain have a formal diagnosis of ADHD [4]. Similarly, in 2015 in the province of Navarre, educational and health records indicate a diagnostic prevalence of around 2.75% of students, based exclusively on accredited medical diagnoses [5]. The fifth edition of the Diagnostic and Statistical Manual of Mental Disorders (DSM-5) of the American Psychiatric Association defines ADHD in children under 17 years of age as the presence of six or more symptoms in one of the domains of inattention or hyperactivity-impulsivity, or in both [6]. Like other psychiatric diagnoses, the aetiology of ADHD is uncertain, and diagnosis is mainly based on clinical signs and cluster of symptoms, as there is no strong evidence of specific biochemical alterations. Therefore, even with potentially clear clinical diagnostic criteria, there is a real risk of symptom overlap with other neurodevelopmental or emotional disorders and, ultimate, misdiagnosis [7]. Also, environmental factors such as socioeconomic status have an impact on psychiatric symptoms.

Despite its high prevalence and long-term implications, there are still gaps in the understanding of how demographic and socio-economic factors influence the diagnosis of ADHD. Several demographic and socioeconomic factors have been associated with the likelihood of being diagnosed with ADHD. Previous studies suggest that low family income is associated with a higher prevalence of ADHD due to factors such as socioeconomic stress [8]. In addition, area of residence (urban, large rural areas or small/medium-sized rural areas) might influence prevalence of ADHD diagnosis, with children in urban areas reported to be more frequently diagnosed than those in rural areas [9].

Migrant status is also considered a relevant factor; children from migrant families face social, cultural and linguistic barriers that may affect diagnosis and treatment but, on other hand, cultural assets, such as diverse perspectives, strong community ties, and unique coping strategies, can also positively influence their care and resilience. Similarly, the type of management of the school the child attends, whether public or charter (i.e. government-funded privately managed), may have a bearing on the diagnosis of ADHD, as these institutions may vary in their tolerance for and approaches to diversity, influencing how cases are identified and referred to physicians [10]. Month of birth has also been linked to ADHD diagnosis, with studies indicating that the youngest children of the school class are more likely to be diagnosed due to differences in maturity with their peers [11, 12].

Gender differences in ADHD diagnosis are well documented, with girls being less likely to be diagnosed [13]. In childhood, the prevalence of ADHD in boys is 2 to 2.5 times higher than that in girls, although in adulthood this ratio becomes closer to equal, since the hyperactivity component generally mitigates with increasing age [14]. In addition, several studies indicate that the number of siblings may influence children’s behaviour and attention, noting differences in the likelihood of being diagnosed when the child has or does not have siblings [15].

In summary, some factors, such as socio-economic status or month of birth, have consistently been shown to be associated with a higher probability of ADHD diagnosis, but this association may vary in different contexts. Other factors such as migrant status, family size, or place of residence have shown mixed results in previous studies regarding their association with ADHD, and others such as educational context have been scarcely studied to date, as has the interaction between these types of socio-demographic factors.

Therefore, the aim of this study is filling these gaps by comprehensively examining possible associations between various demographic and socioeconomic variables and ADHD diagnosis. Specifically, the study will assess whether the observed associations of two factors— quarter of birth and migration status—were subject to modification by type of school attended (public vs. charter). We will analyze this variable in depth to explore the potential modulation it may have on the effects of relative age and migrant status on the probability of ADHD diagnosis. Understanding these associations is crucial to identifying the variations, diversity, and lack of homogeneity in current patterns of diagnosis. By providing new insights into these factors, we hope to contribute to the development of strategies based on the best scientific evidence in the highly controversial field of ADHD.

## Materials and methods

### Study design and data source

A population-based case-control study nested in a cohort was carried out. Data were obtained from the databases of the Navarre Department of Education (EDUCA) and the Navarre Health Service (BARDENA) [16]. The linkage between these sources was performed by the personnel responsible for pseudonymizing health data for secondary use in BARDENA, ensuring that the researchers received fully anonymized data. To resolve possible inconsistencies in the data, decision algorithms and automated probabilistic techniques were implemented to ensure the integrity and accuracy of the combined information.

### Population and follow-up

In 2019, the total population of Navarre was approximately 654 000 inhabitants, of whom an estimated 142.000 (around 22%) were children and adolescents (20 years old or less) [17]. The study included individuals born between 1991 and 2011 who had access to public health assistance by the Navarre Health Service and who attended at least one year of compulsory education between 2007 and 2017 in schools funded by the Government of Navarre, whether public or charter schools (during this period, there was only one private, non-subsidised school in the region). Students were excluded if they had health insurance other than that of the National Health System, such as that provided by some public mutual insurance companies. Follow-up of participants started from the age of 5 years and continued until completion of compulsory education or loss of availability of health data, whichever occurred last. Follow-up was discontinued at the latest in case of death, when reaching 20 years of age or on 20 November 2019, the closing date of the study. The age span 5–20 was selected because it is the age at which children may be attending primary or secondary school in Spain. The incidence of ADHD is studied in the period from 2003 to 2019. A more detailed description of the cohort is described elsewhere [3].

### ADHD diagnostic codes and study variables

Individuals with a diagnosis of ADHD during the study period were considered cases. ADHD diagnoses recorded both in public Primary Care in Navarre, using the International Classification of Primary Care-2 (ICPC-2), and in public Specialised Care in Navarre, using the 10th Revision of the International Classification of Diseases (ICD-10) were included (Table S1). Two reviewers (LL and LCS) validated all ADHD diagnoses by reviewing the literal texts associated with ICPC-2 codes P81, P22, P23 and P24. Items other than attention deficit, hyperactivity, ADHD or clear synonyms, were excluded, as well as those showing uncertainty on the ADHD diagnosis, by including terms such as ‘suspected’, ‘possible’, ‘concern’, or question marks. Each reviewer initially validated half of the database, and doubtful results were subsequently reviewed by a second reviewer. Disagreements were solved by consensus. The date of diagnosis was established as the first occurrence of the ADHD record in either Primary or Specialised Care.

In the current study, several variables were used to analyze the demographic and socioeconomic factors that could be related to ADHD diagnosis. The considered variables were gender, date of birth, country of origin of both children and parents, family income, place of residence, number of siblings (twins are treated like regular siblings), and type of school the children attended, distinguishing between public and charter schools. These variables provide a broader view to explore possible correlations between demographic and socioeconomic factors and the diagnosis of ADHD in the population.

### Statistical analysis

Categorical variables were described using frequencies and percentages. Quantitative variables were summarized using mean and standard deviation (SD). Differences between groups on categorical variables were assessed using the χ² test. Differences in continuous variables were determined using Student’s t-test or the Mann-Whitney test, depending on the normality of the data.

For data analysis, a multivariate conditional logistic regression was performed. This statistical technique was used to assess associations between ADHD diagnosis (dependent variable) and various demographic and socio-economic variables (independent variables), such as family income, place of residence, migration status, month of birth, number of siblings and type of school. The model allowed adjustment for these variables, providing adjusted odds ratios (ORs) and 95% confidence intervals (95% CIs) for each association. This allowed us to identify which factors were independently associated with ADHD diagnosis in the study population. Independent variables were described as follows:

- Income data: Dichotomous (<€18,000/≥€18,000) based on the child’s assigned pharmaceutical co-payment group, derived from 2018 taxable income. This value reflects the individual taxable income of one contributing parent (in individual returns) or, less commonly, the total household income (in joint returns). It serves as a pragmatic, individual-level proxy for the child’s familial socioeconomic context.
- Housing was classified in urban, large rural areas or small/medium-sized rural areas according to population size. Specifically, areas with less than 2,500 inhabitants were categorised as small/medium sized rural areas; areas with between 2,500 and 10,000 inhabitants were categorised as large rural areas; and areas with more than 10,000 inhabitants were categorised as urban.
- Migrant status was divided in four groups: (a) born in Spain with both parents Spanish; (b) born abroad; (c) born in Spain to non-Spanish parents; (d) country of birth unknown.
- According to the month of birth, children born in January-March were compared with those born in April-June, July-September and October-December, respectively. In Spain, school enrolment is based strictly on the calendar year a child turns six, so children born in January are the oldest and those born in December are the youngest in their class.
- According to the number of siblings, single children were taken as reference and compared with (a) children with one or two siblings; and (b) children with three or more siblings.
- Schools were classified based on their funding and management structures: those that are entirely funded and operated by the government, known as public schools; and those that receive government funding but are privately managed, referred to as charter schools.

We hypothesized that the relationship between quarter of birth and migration status with ADHD diagnosis could be influenced by type of school (public vs. charter). To test this, interaction terms were included in the model. We report main effects for variables without interactions and conditional effects for all categories involved in the interaction. The latter were obtained by reparameterizing the model and rotating the reference categories.

The main analysis compared the risk of ADHD diagnosis among children and adolescents who met certain exposure factors versus those who did not. Cases included in this analysis were those patients with an ADHD diagnosis that were clinically validated.

Analyses were carried out with the statistical software R (Rstudio, R version 4.3.3 (2024-02−29)) [18].

### Sample selection

The sample selection was performed using incidence density (risk-set) sampling. For each incident ADHD case, three controls were selected from population cohort members at risk (free of ADHD) at the case’s diagnosis date (index date), matched on sex and year of birth.

This 1:3 sampling maximises statistical power, improves precision and ensures that odds ratios from conditional logistic regression unbiasedly estimate incidence rate ratios. Matching on sex and birth year, combined with risk-set sampling, controls for these factors and for calendar time. The matching procedure was implemented with the ccwc function from the Epi package in R.

The observation period ended with the occurrence of the event of interest (ADHD) or censoring of the participant. The analysis established a common temporal origin for all subjects, which allowed for standardisation of exposure time.

### Study approval

The study protocol was approved by the Ethics Committee of the Government of Navarre, Spain in 2016 (project 2016/73). All procedures were in accordance with the ethical standards of the institutional committee.

## Results

### Description of the study cohort

**Fig. 1:**
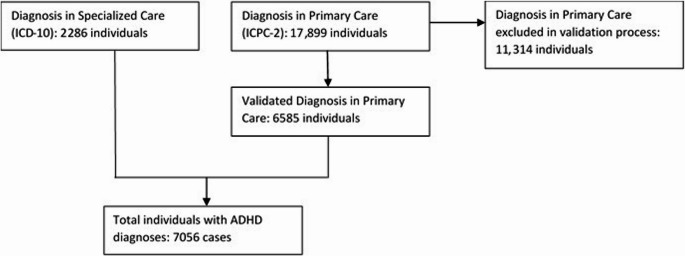
ADHD case selection process (*an individual may have more than one diagnostic code during the follow-up period)

The study cohort consisted of 124,582 children and adolescents (1,167,478 person-years). Of these, 7,056 were patients with a diagnosis of ADHD (cases), resulting in an overall incidence rate of 6.04 cases per 1000 person-years, and 21,168 subjects were selected as controls. The ADHD case selection process flow is shown in Fig. 1.

The characteristics of the analyzed variables can be observed in Table 1.

**Table 1:** Characteristics of the study cohort

		Controls (*N* = 21,168)	Cases (*N* = 7,056)
Gender	Female (%) Male (%)	5,844 (27.6) 15,324 (72.4)	1,948 (27.6) 5,108 (72.4)
Year of birth	Year of birth, mean (SD)	2,001.3 (4.6)	2,001.3 (4.6)
Income	< 18,000 euros/year (%) ≥ 18,000 euros/year (%)	11,953 (56.7) 9,215 (43.3)	4,111 (58.3) 2,945 (41.7)
Migration status	Born in Spain, Spanish parents (%)	15,961 (75.4)	5,782 (81.9)
	Born abroad (%)	2,702 (12.8)	486 (6.9)
	Born in Spain, non Spanish parents (%)	1,869 (8.8)	573 (8.1)
	‍Unknown (%)	636 (3.0)	215 (3.0)
Place of residence	Urban (%)	10,373 (49)	3,742 (53)
	Large rural areas (%)	6,597 (31.2)	1,983 (28.1)
	Small/medium sized rural areas (%)	2,843 (13.4)	942 (13.4)
	Unknown (%)	1,355 (6.4)	389 (5.5)
Quarter of birth	January-March (%)	5,238 (24.7)	1,357 (19.2)
	April-June (%)	5,530 (26.1)	1,721 (24.4)
	July-September (%)	5,462 (25.8)	1,871 (26.5)
	October-December (%)	4,938 (23.3)	2,107 (29.9)
Number siblings	Three or more siblings (%)	4,004 (18.9)	1,166 (16.5)
	One or two sibling (%)	11,054 (52.2)	3,681 (52.2)
	Only child (%)	6,110 (28.9)	2,209 (31.3)
Type of school	Charter school (%) Public schools (%)	6,820 (32.2) 14,348 (67.8)	2,754 (39.0) 4,302 (61.0)

Distribution between male/female for other examined aspects is shown in Table S2.

As expected by the matching technique, the percentage of females was similar (27.6%) in cases and controls. Also, the mean age of the participants, expressed as the mean year of birth, was similar in both groups (2,001).

The analysis of family income showed that 58.3% of the cases belonged to households with an income of less than 18,000 euros per person and year, compared to 56.7% of the controls.

The place of residence also showed notable differences between the groups. More than half of the population in both groups resided in urban areas; however, a higher percentage of cases lived in urban environments compared to controls (56.1% vs. 52.4%). Conversely, more controls lived in large rural areas than cases (29.7% vs. 26.5%). In small or medium sized rural areas, no differences were observed between the groups.

In terms of migrant status, the majority of cases (81.9%) were patients born in Spain with both parents also born in Spain, compared to 75.4% of controls. Additionally, a smaller percentage of cases (6.9%) were born abroad, in contrast to 12.8% of controls. Individuals born in Spain but with at least one parent born abroad, represented 8.1% of cases and 8.8% of controls. Unknown status for parental origin accounted for 3% in both groups.

Significant differences were observed between groups in the distribution of month of birth. Births in the quarters April to June and October to December were more frequent among cases (26.5% and 29.9%, respectively), while among controls, most births occurred between January and March (24.7%) and April and June (26.1%).

Variations in the number of siblings between cases and controls were also observed. A lower percentage of cases had three or more siblings (16.5%) compared to controls (18.9%), while the proportion of only children was slightly higher among cases (31.3%) than among controls (28.9%).

Finally, a significantly higher percentage of cases attended charter schools (39.0%) compared to controls (32.2%).

### Results on the association between socioeconomic and demographic factors and ADHD diagnosis

Multivariate logistic regression analysis reveals a number of statistically significant associations between various socio-demographic variables and ADHD diagnosis. The results are shown in Table 2.

**Table 2:** Results on the association between socioeconomic and demographic factors and ADHD diagnosis

Variable (reference)	Category	Odds Ratio	95% CI	*p*-value
Main Effects				
**Income** (≥ 18,000 euro/person/year)	< 18,000 euros/person/year	1.23	1.15–1.31	< 0.001
**Quarter of birth** (January-March)	April-June	1.20	1.11–1.32	< 0.001
	July-September	1.35	1.24–1.47	< 0.001
	October-December	1.69	1.55–1.83	< 0.001
**Place residence** (Urban)	Large rural	0.88	0.82–0.93	< 0.001
	Small/medium rural	0.94	0.86–1.02	0.135
**Number of siblings** (only child)	3 or more	0.83	0.76–0.91	< 0.001
	One or two	0.92	0.86–0.99	0.014
**Conditional Effects (Interactions)**				
**Migration status** (Ref: Born in Spain, Spanish parents)	**In Public schools**			
Born abroad		0.40	0.36–0.46	< 0.001
Spain, non-Spanish parents		0.79	0.70–0.89	< 0.001
Unknown		0.90	0.23–3.48	0.819
	**In Charter schools**			
Born abroad		0.77	0.63–0.94	0.010
Spain, non-Spanish parents		1.04	0.81–1.33	0.740
Unknown		0.84	0.70–0.99	0.041
**School type** (Ref: Public)	**By migration status**			
Charter vs. Public	Born in Spain, Spanish parents	1.21	1.13–1.30	< 0.001
Charter vs. Public	Born abroad	2.32	1.84–2.92	< 0.001
Charter vs. Public	Born in Spain, non-Spanish	1.60	1.23–2.08	< 0.001
Charter vs. Public	Unknown	1.13	0.29–4.40	0.861

Household income was significantly associated with the odds of having a recorded ADHD diagnosis. Children from families with an income of less than < 18,000 euros per person and year are 23% more likely to be diagnosed with ADHD (OR = 1.23, 95% CI: 1.15–1.31) than families with ≥ 18,000 euros per person and year.

With respect to month of birth, significant associations with the likelihood of receiving an ADHD diagnosis were found. Children born between April and June were 20% more likely to be diagnosed with ADHD compared to those born between January and March (OR = 1.20, 95% CI: 1.11–1.32). For those born between July and September, the risk was even higher, with a 35% increase in the probability of diagnosis (OR = 1.35, 95% CI: 1.24–1.47). The strongest association was observed in those born between October and December, who are 69% more likely to be diagnosed with ADHD (OR = 1.69, 95% CI: 1.55–1.83).

Place of residence also showed a significant association with recorded ADHD diagnosis. Children living in large rural areas were 12% less likely to be diagnosed with ADHD compared to those living in urban areas (OR = 0.88, 95% CI: 0.82–0.93). On the other hand, no significant differences were found for those living in small/medium sized rural areas (OR = 0.94, 95% CI: 0.86–1.02) compared to urban areas.

Regarding the number of siblings, having three or more siblings was associated with a 17% lower odds of receiving an ADHD diagnosis (OR = 0.83, 95% CI: 0.76–0.91), compared to being an only child. Having one or two siblings was also associated with a slightly lower odds of diagnosis by 8% (OR = 0.92, 95% CI: 0.86–0.99).

The interaction between quarter of birth and type of school was not statistically significant, indicating that the association between quarter of birth and recorded ADHD diagnosis did not differ by school type. Therefore, this interaction term was removed from the final model.

**Fig. 2:**
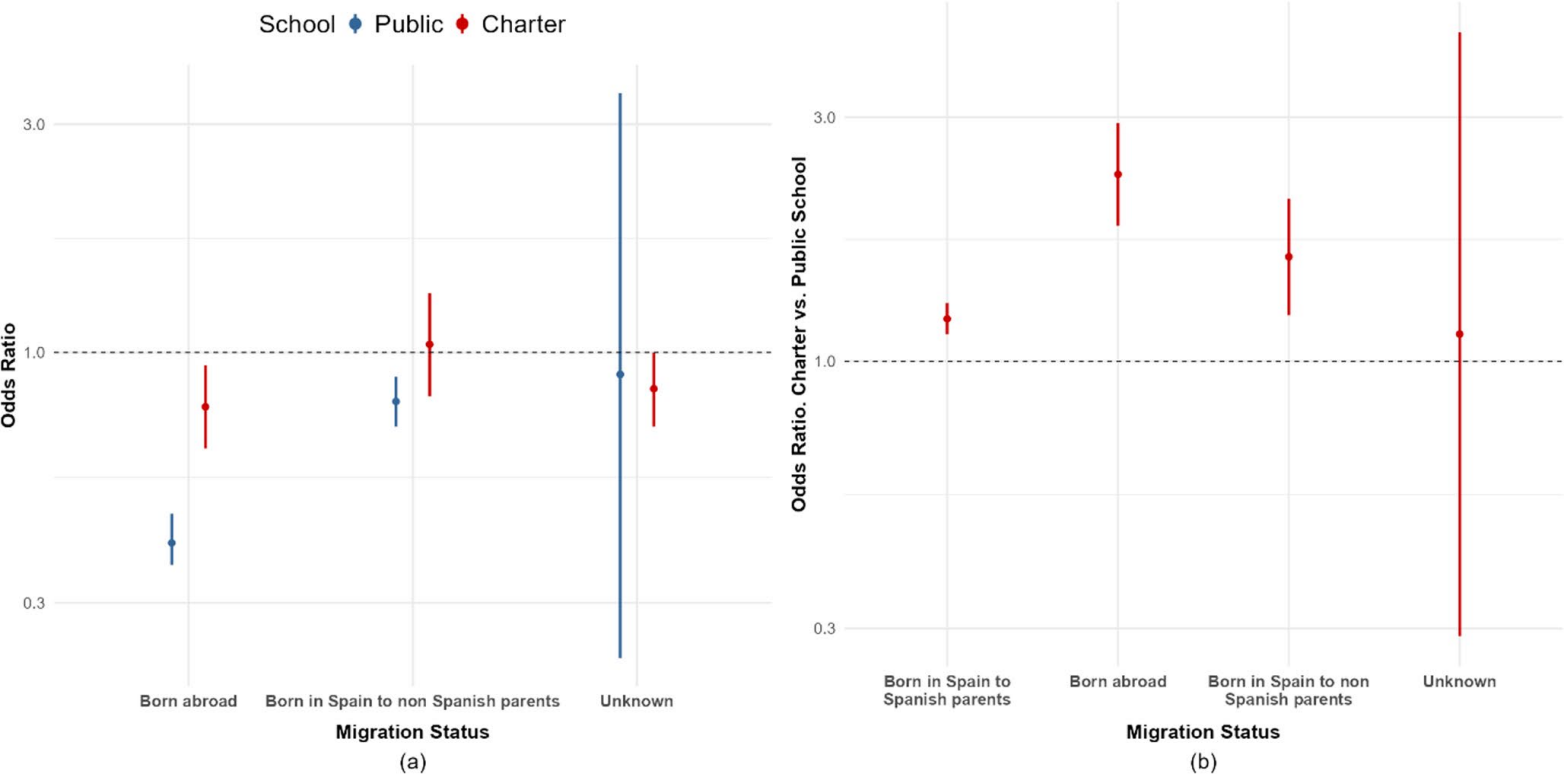
a. Adjusted odds ratios (OR) of ADHD diagnosis for migration status *by type of school*. The reference group is children born in Spain to Spanish parents. **b**. Adjusted odds ratios (OR) of ADHD diagnosis for type of school *by migration status*. The reference group is public schools

A significant interaction was observed between migrant status and school type. On the one hand, within those attending public schools, children born abroad were 60% less likely to be diagnosed with ADHD compared to those born in Spain to Spanish parents (OR = 0.40, 95% CI: 0.36–0.46). Those born in Spain to non-Spanish parents also had a 21% lower odds of diagnosis than those born in Spain to Spanish parents (OR = 0.79, 95% CI: 0.70–0.89). However, no significant association was found in those with an unknown migration background (OR = 0.90, 95% CI: 0.23–3.48). On the other hand, within those attending charter schools and compared to those born in Spain to Spanish parents, children born abroad were 23% less likely to be diagnosed with ADHD (OR = 0.77, 95% CI: 0.63–0.94), no significant differences were found with children born in Spain to non-Spanish parents (OR = 1.04, 95%CI: 0.81–1.33), and those with an unknown migration background were 16% less likely to be diagnosed with ADHD (OR = 0.84, 95% CI: 0.70–0.99) (Fig. 2.a).

Among children born in Spain to Spanish parents, the odds of diagnosis with ADHD was 21% higher for those attending charter schools (OR = 1.21, 95% CI: 1.13–1.30). In contrast, among children born abroad, those in charter schools had more than twice the odds of receiving an ADHD diagnosis compared with those attending public schools (OR = 2.32, 95% CI: 1.84–2.92). For children born in Spain to non-Spanish parents, charter school attendance was associated with 60% higher odds of diagnosis (OR = 1.60, 95% CI: 1.23–2.08). No significant differences were observed among children with an unknown background (OR = 1.13, 95% CI: 0.29–4.40) (Fig. 2.b).

## Discussion

This study suggests that demographic, socio-economic and educational settings significantly influence the likelihood of receiving a recorded ADHD diagnosis in our analyzed population, which is in line with previous studies.

In terms of socio-economic status, children from families with an income of less than 18,000 euros per person and year showed higher odds of being diagnosed with ADHD. This is consistent with what has been shown in other studies [19, 20]. Rather than reflecting differences in the underlying prevalence of ADHD, this association might reflect how economic factors affect family environment and living conditions, which could contribute to increased stress and exacerbation of ADHD symptoms [21].

In terms of month of birth, the results reinforce the hypothesis that children born in the last months of the year are more likely to receive an ADHD diagnosis. This trend aligns with the ‘relative age effect’, which refers to the impact of a child’s age relative to their peers within the same educational cohort. School enrolment based on the calendar year in which the child reaches school age means that those born at the end of the year are the youngest in their class, making them nearly a year younger than some classmates. This age gap can result in noticeable differences in behavior and maturity [12, 22]. Younger children in a classroom setting may display behaviors such as inattention, hyperactivity, or impulsivity—traits often associated with ADHD. However, these behaviors might stem from developmental immaturity rather than a neurodevelopmental disorder, increasing the likelihood of referral and diagnosis [12, 23–25].

The results suggest that place of residence is associated with differences in ADHD diagnostic rates, being less likely in children living in large rural areas compared to those in urban areas. Other studies have also found that children living in small/medium sized rural areas were less likely to be diagnosed with ADHD [9]. Factors such as higher exposure to nature or lifestyle have been associated with reduced ADHD diagnoses and symptom severity [26]. In addition, in urban areas there is greater awareness and resources for diagnosis, which could contribute to higher rates of ADHD labelling.

Family size was also associated with lower odds of receiving an ADHD diagnosis. The results show that children with three or more siblings have a lower risk of being diagnosed with ADHD, suggesting that family dynamics could play a role in the manifestation of ADHD symptoms, the family tolerance towards them or the health care seeking behaviour. This trend is also observed, albeit more modestly, in those with one or two siblings. Children could benefit from greater social support at home and less attention focused on individual behaviors, which could reduce the identification of ADHD symptoms. Controversial results have been observed on this issue [15, 27, 28].

The analysis of migration background alongside the school’s type reveals that children born abroad who attend public schools show a substantially lower likelihood of receiving a diagnosis compared to those born in Spain to Spanish parents. This pattern was also observed, although to a lesser degree, among children born in Spain to non-Spanish parents. However, these differences were reduced or disappeared entirely in charter schools. In the latter, although children born abroad continued to have lower odds of being diagnosed than their Spanish-born counterparts, the magnitude of the difference was smaller, and no statistically significant differences were found for children born in Spain to non-Spanish parents. These results are consistent with other studies, although the relationship remains controversial, and mixed findings have been reported in different contexts [29–32]. This observation raises questions about the ways in which cultural and social contexts may influence the perception and diagnosis of symptoms. Cultural differences in the recognition and management of childhood behavior, along with potential barriers to accessing mental health services, could explain the lower rates of diagnosis in these populations [33].

When comparing the probability of diagnosis between public and charter schools, children born abroad and those born in Spain to non-Spanish parents were more likely to receive an ADHD diagnosis in charter schools. Possible explanations would include the greater availability of resources in charter schools to evaluate and facilitate medical diagnoses, or educational expectations oriented toward strict behavioral standards and academic performance, among others [34]. The type of school is a factor that has been scarcely studied to date; however, as these findings suggest, it may have an important impact on the ADHD diagnosis [35].

As stated in the introduction, there is a genuine risk of misdiagnosis due to the uncertainty surrounding ADHD etiology and the absence of specific biological markers. The disparities observed in our study variables may reflect not only differences in the manifestation or intensity of symptoms but also differences in how adults (professionals, family members, teachers, school counsellors) interpret and respond to certain childhood behaviors, and in pathways to assessment: teacher referral practices, parental help-seeking, language barriers, school counsellor availability, and proximity to specialist services. In this regard, sociodemographic factors may contribute to both overdiagnosis and underdiagnosis, depending on cultural expectations, access to healthcare, and the specialized training of those evaluating children.

Rather than indicating challenges of applying standardized diagnostic criteria across contexts, these findings highlight the complexity of applying these criteria across diverse social and cultural contexts. While the DSM-5 provides detailed clinical criteria, the fact that socioeconomic and educational factors can be so influential may pose a challenge to the objectivity of diagnosis, opening the door to diverse interpretations of the same symptoms. Therefore, it is crucial to further investigate the validity and reliability of assessment tools across different social and cultural contexts, as well as to promote professional and educational training aimed at ensuring more accurate and earlier detection of those cases that genuinely require intervention.

Taken together, our findings underscore the need to continue examining how economic, cultural, and educational inequalities might shape patterns of recorded ADHD diagnosis. Identifying and addressing these determinants is essential to prevent inappropriate diagnoses, ensure equitable care, and enhance the quality of life for children and adolescents exhibiting attention and hyperactivity difficulties.

The study has several significant strengths. The large sample size, including 28,224 participants, provides adequate statistical power to detect significant associations. Adopting a case-control ratio of 1:3 not only strengthens the robustness of the comparative analysis, but also optimises available resources by focusing the study on a sample size that adequately balances statistical precision and practical feasibility of the project [36]. The use of real world data obtained from education and healthcare system databases provides a realistic and comprehensive view of the population studied from different perspectives. Multivariate analysis, adjusting for multiple socio-demographic variables, allows for a more detailed understanding of the factors associated with ADHD diagnosis. Independent validation of ADHD diagnoses by two raters increases the reliability of the data. In addition, consideration of multiple factors, including income, place of residence, migrant status, month of birth, number of siblings and type of school, allows for a comprehensive analysis. Identifying interactions, such as with migration status and school type, provides more in-depth information on how these combined factors affect ADHD diagnosis.

The study has certain limitations that need to be considered. The study has a retrospective and observational design, and thus it is highly dependent on the quality and accuracy of existing records, which may introduce errors or incomplete information affecting the results. Income data used were limited, relying on the pharmaceutical contribution group as a proxy measure, which may not fully reflect the socio-economic situation of families. The wide confidence intervals observed for the ‘unknown’ migrant status category reflect the limited statistical precision resulting from the small number of observations in this group. The effect of time is another factor, as diagnostic criteria and awareness of ADHD may have changed during the study period, affecting diagnosis rates. Importantly, a number of plausible confounders were not included, such as parental education, parental mental health/ADHD, health-care utilisation, school performance/special educational needs, and neighbourhood deprivation. The results are based on a specific region of Spain (Navarre), so the conclusions may not be applicable to other regions or countries with different socio-demographic characteristics and educational and healthcare systems.

The diagnosis of ADHD may present ambiguities due to variability in the criteria employed, as well as differences in the clinical interpretation of symptoms. The absence of a universally accepted and rigorously established definition may result in the claimed disorder not being fully accurately identified, which in turn affects the validity of the conclusions.

In summary, the study enhances current knowledge by demonstrating the significant impact of socioeconomic factors on ADHD diagnosis rates. It reveals that children from lower-income families, those attending charter schools, those living in urban areas and those born in the last months of the year have higher odds of receiving an ADHD diagnosis, whereas children with a migrant background and those with the largest number of siblings showed lower odds of diagnosis. These findings highlight the central role of social, cultural and educational context in shaping assessment pathways and diagnostic practices, reinforcing the need for cautious interpretation of ADHD diagnostic patterns and for strategies aimed at reducing inequities in identification and care.

## Supplementary Information

Below is the link to the electronic supplementary material.

ESM 1 (DOCX 20.0 KB)

## Data Availability

Data were obtained from the Navarre Health Service and the Education Department in Navarre. Restrictions apply to the availability of these data.
