# Supplementary material for: Demographic and socioeconomic factors influencing ADHD diagnosis in children and adolescents: a population-based nested case–control study
Source: Eur Child Adolesc Psychiatry. 2026 Mar 16;35(6):1947–56. doi: 10.1007/s00787-026-03007-5 (PMC13337746; doi:10.1007/s00787-026-03007-5)
Supplement: Supplementary file 1 — (DOCX 20.0 KB) [file 787_2026_3007_MOESM1_ESM.docx]

Table S1. Attention deficit hyperactivity disorder (ADHD) diagnostic codes

| **Classification system** | **ADHD diagnostic code** |
| --- | --- |
| International Classification of Primary Care (ICPC) | P81: Hyperactivity disorder (a) |
|  | P22: Behavioral signs/symptoms in children. Behavioral or conduct problems(a) |
|  | P23: Behavioral signs/symptoms in adolescents(a) |
|  | P24: Specific learning problems(a) |
| 10th Revision of the International Classification of Diseases (ICD-10) | F90.0: Attention deficit hyperactivity disorder, with predominance of inattentive type |
|  | F90.1: Attention deficit hyperactivity disorder, with predominance of hyperactive type |
|  | F90.8: Attention deficit hyperactivity disorder, other type |
|  | F90.9: Attention deficit hyperactivity disorder, type not specified |

(a) All P81, P22, P23 and P24 literals were manually reviewed by two researchers (LL and LC). Only ADHD validated cases were finally included, after exclusion of literals outside the scope of ADHD or showing uncertainty on the ADHD diagnosis, by including terms such as ‘suspected’, ‘possible’, ‘concern’, or question marks.

Table S2. Characteristics of the study cohort, stratified by gender.

|  |  | FEMALE  7,792 (27.6) | | MALE  20,432 (72.4) | |
| --- | --- | --- | --- | --- | --- |
|  |  | Controls  5,844 (75) | Cases  1,948 (25) | Controls  15,324 (75) | Cases  5,108 (25) |
| Year of birth | Year of birth, mean (SD) | 2,001.65 (4.36) | 2,001.65 (4.36) | 2,001.23 (4.74) | 2,001.23 (4.74) |
| Income | ≤ 18,000 euros/year (%)  ≥18,000 euros/year (%) | 3,225 (55.4)  2,619(44.6) | 1,083 (55.6)  865 (44.4) | 8,728 (57.2)  6,596 (43.8) | 3,028 (59.4)  2,080 (40.6) |
| Migration status | Born in Spain, Spanish parents (%) | 4,434 (75.9) | 1,589 (81.6) | 11,527 (75.2) | 4,193 (82.1) |
|  | Born abroad (%) | 719 (12.3) | 152 (7.8) | 1,983 (12.9) | 334 ( 6.5) |
|  | Born in Spain, non Spanish parents (%) | 510 ( 8.7) | 146 (7.5) | 1,359 (8.9) | 427 (8.4) |
|  | ‍Unknown (%) | 181 ( 3.1) | 61 (3.1) | 455 (3.0) | 154 (3.0) |
| Place residence | Urban (%) | 2,871 (49.1) | 1,069 (54.9) | 7,502 (49) | 2,673 (52.3) |
|  | Large rural areas (%) | 1,821 (31.1) | 505 (25.9) | 4,776 (31.2) | 1,478 (28.9) |
|  | Small/medium sized rural areas (%) | 810 (13.8) | 270 (13.9) | 2,033 (13.3) | 672 (13.2) |
|  | ‍Unknown (%) | 342 (5.9) | 104 (5.3) | 1,013 (6.6) | 285 (5.6) |
| Quarter of birth | January-March (%) | 1,450 (24.8) | 340 (17.5) | 3,788 (24.7) | 1,017 (19.9) |
|  | April-June (%) | 1,532 (26.2) | 446 (22.9) | 3,998 (26.1) | 1,275 (25.0) |
|  | July-September (%) | 1,499 (25.7) | 543 (27.9) | 3,963 (25.9) | 1,328 (26.0) |
|  | October-December (%) | 1,363 (23.3) | 619 (31.8) | 3,575 (23.3) | 1,488 (29.1) |
| Number siblings | Three or more siblings (%) | 1,077 (18.4) | 320 (16.4) | 2,927 (19.1) | 846 (16.6) |
|  | One or two sibling (%) | 3,114 (53.3) | 1,016 (52.2) | 7,940 (51.8) | 2,665 (52.2) |
|  | Only child (%) | 1,653 (28.3) | 612 (31.4) | 4,457 (29.1) | 1,597 (31.3) |
| Type of school | Charter school (%)  Public schools (%) | 1,959 (33.5)  3,885 (66.5) | 854 (43.8)  1,094 (56.2) | 4,861 (31.7)  10,463 (68.3) | 1,900 (37.2)  3,208 (62.8) |
